# The long-term course of fatigue following breast cancer diagnosis

**DOI:** 10.1186/s41687-020-00187-9

**Published:** 2020-05-18

**Authors:** Karin Biering, Morten Frydenberg, Helle Pappot, Niels Henrik Hjollund

**Affiliations:** 1 Danish Ramazzini Centre, Department of Occupational Medicine, University Research Clinic, Regional Hospital West Jutland, Herning, Denmark; 2 Section of Biostatistics, Department of Public Health, Aarhus University, Aarhus, Denmark; 3 Department of Oncology, Rigshospitalet, University Hospital of Copenhagen, Copenhagen, Denmark; 4 AmbuFlex/WestChronic, Occupational Medicine, University Research Clinic, Regional Hospital West Jutland, Herning, Denmark; 5 Department of Clinical Epidemiology, Aarhus University Hospital, Aarhus, Denmark

**Keywords:** Mammography, Breast Cancer, Fatigue, Longitudinal, Rehabilitation, Long-term

## Abstract

**Purpose:**

Fatigue following breast cancer is a well-known problem, with both high and persistent prevalence. Previous studies suffer from lack of repeated measurements, late recruitment and short periods of follow-up. The course of fatigue from diagnosis and treatment to the long-time outcome status is unknown as well as differences in the level of fatigue between treatment regimens. The purpose of this study was to describe the long-time course of fatigue from the time of clinical suspicion of breast cancer, its dependence of patient characteristics and treatment regimens and the comparison with the course of fatigue among women with the same suspicion, but not diagnosed with breast cancer.

**Methods:**

Three hundred thirty-two women referred to acute or subacute mammography was followed with questionnaires from before the mammography and up to 1500 days. Fatigue was measured by the Multidimensional Fatigue Inventory (MFI-20). The women reported their initial level of fatigue before the mammography and thus without knowledge of whether they had cancer or not. Both women with and without cancer were followed. Women with cancer were identified in the clinical database established by Danish Breast Cancer Cooperative Group (DBCG) to collect information on treatment regimen.

**Results:**

Compared to fatigue scores before diagnosis, women with breast cancer reported a large increase of fatigue, especially in the first 6 months, followed by a slow decrease over time. Despite the long follow-up period, the women with breast cancer did not return to their level of fatigue at time of the mammography. Women without breast cancer, experienced a rapid decrease of fatigue after disproval of diagnosis followed by a steadier period.

**Conclusions:**

Fatigue is a persistent problem in women diagnosed with breast cancer, even several years following diagnosis and treatment. The women with breast cancer were most affected by fatigue in the first 6 months after diagnosis.

## Introduction

Fatigue following breast cancer is a well-known and common problem, both during treatment and in the rehabilitation period. The occurrence of fatigue ranges from 35% to almost all women in different studies [1, 2]. A large proportion of patients consider fatigue as their main problem [3–8] with large consequences for the women’s quality of life and rehabilitation. Fatigue is even suggested to be an independent marker of long-term survival without recidivism [9].

The pathophysiological background for fatigue is to a large extend unknown and correlations to various biomarkers are weak and inconsistent [3]. The pathways leading to fatigue following breast cancer are suggested to relate to the disease itself, to other diseases, to the treatment or to psychological reaction on the disease [2, 10]. When fatigue is occurring and when it is most severe is thus unknown, however, the highest degree of fatigue seems to occur in the first half year after treatment completion [11, 12].

Fatigue often persist further than the treatment period, but previous studies have focused mostly on the treatment period only, and in most cases with use of only one or two measurement points, which make a description of the actual course of fatigue impossible or simplistic [13]. Studies on long-term fatigue usually use only one follow-up measurement and compared to healthy populations, increased prevalence and intensity of fatigue is found, even years after treatment [14–16].

Thus, studies describing the course of fatigue before the beginning of treatment and in detail over time are rare, and knowledge about this is warranted to be able to inform women with breast cancer about the expected development of fatigue. Two exceptions are Salmon et al. who measured fatigue 10 times over a 2-year period [12], and Bower et al. who measured fatigue up to 7 times over 4 years [17], however both studies were without a comparison group.

The purpose of this study was to describe the course of fatigue following breast cancer and its association to patient characteristics in terms of treatment regimens and compare with the course of fatigue among women referred to the same diagnostic procedure, but not subsequently diagnosed with breast cancer.

## Methods

The source population was women referred to mammography at the Danish public hospital, Randers Regional Hospital, during the period October 2004 to May 2006. The catchment area of the hospital includes rural locations as well as the fifth largest city in Denmark (around 62.000 inhabitants) [18]. Patients were referred by their family doctor. Based on an evaluation of risk, a consultant at the Department of Radiology assigned the referred patients to one of three categories: acute (within 1–2 weeks), subacute (within 2–3 weeks) or not acute (4 weeks or more). The procedure is described in detail elsewhere [19].

### Inclusion and follow-up

We included women in the acute and subacute group aged below 67 years, who did not have a history of previous breast cancer. Women referred to a non-acute mammography was excluded. The women received a baseline questionnaire by mail before the date of their mammography. The women were asked to complete and return the questionnaire in a prepaid envelope or to answer the same questionnaire online. Non-respondents in both groups were mailed a reminder after 10 days, given that the date of their mammography was not reached. A few women returned the questionnaire after the mammography. If the questionnaire was filled in after the mammography took place, the woman was excluded, due to the risk of her knowing the result of the mammography (*n* = 11).

The women were followed with questionnaires every 3 months, irrespectively of whether they were diagnosed with breast cancer or not. The women could at any time choose their preferred method of answering the questionnaires; either by paper or web. The latter was done by providing their e-mail address in the questionnaire and if so the following questionnaires were sent out via an email including a link. The women, who did not respond on follow-up questionnaires, were mailed a reminder after 17 and 34 days.

Due to economic reasons, women without breast cancer were only followed for more than 1 year (four questionnaires) if they answered by the web method. Collection of data continued until December 2013. During follow-up some women left the study and after 5 years follow-up, only 96 women (30%) still answered questionnaires Due to this, we limited the analysis to 1500 days of follow-up.

**Fig. 1:**
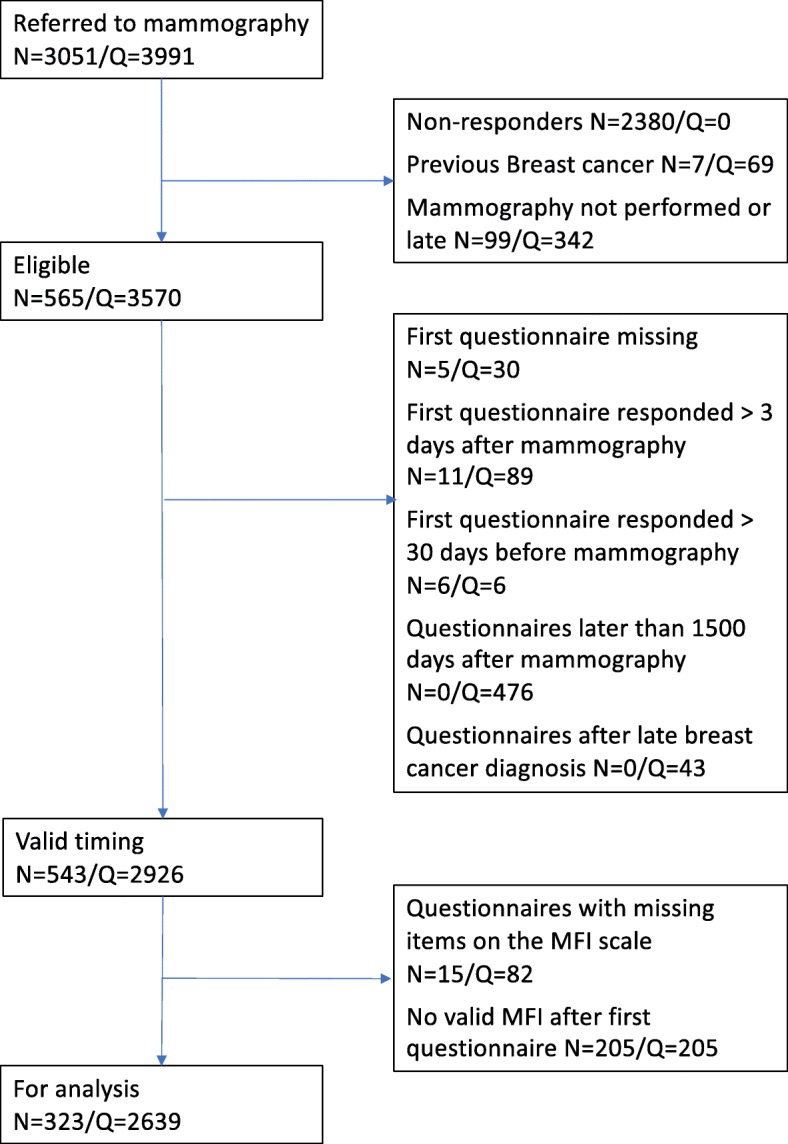
Flowchart of inclusion of participants and questionnaires in a study of long-term fatigue after breast cancer

The flowchart illustrates the recruitment process, with different types of exclusions of both participants (N) and questionnaires (Q) (Fig. 1). Due to the design with repeated measurements, each woman (N) could have more than one questionnaire (Q) and thus excluding women also resulted in exclusion of questionnaires provided by the those women. An exception was the decision to include only data from the first 1500 days of the study. In that case 476 questionnaire was not used, but the women remained in the study with all previous measurements. The final sample for analysis was 323 women with 2639 questionnaires.

### Questionnaires

The baseline questionnaire contained questions about general health, anxiety and depression, fatigue, as well as education, work and family situation. Fatigue was measured in both the baseline questionnaire and in the follow-up questionnaires by the Multidimensional fatigue inventory (MFI) [20, 21], which consists of 20 items and, by which 5 dimensions and a total sum score can be calculated. Only the total sum score was used in this paper. All questionnaires inquired also on general health (GH) from SF36 [19, 22] and anxiety and depression (HADS) [23]. Furthermore, women with breast cancer were asked about sequelae such as pain, swelling and sensitivity disturbances in the follow-up questionnaires. Sum-scores were based on the mean value of non-missing items, if no more than half of the items were missing, as suggested by Bell et al. 2016 [24].

### Other data sources

In Denmark, every resident is provided with a permanent and unique civil registration number that enables individual-level linkage between registers [25]. We utilised this to obtain further information about the women using three Danish registers: The Danish Breast Cancer Cooperative Group, The Danish National Patient Registry and Danish Register for Evaluation of Marginalisation.

The Danish Breast Cancer Cooperative Group (DBCG) was established in 1976 with the aim of monitoring diagnostic procedures and treatment of breast cancer on a nation-wide basis [26]. We specifically used the information on adjuvant treatment regime. The women were categorized as high-risk patients (age ≤ 35 years or tumour > 2 cm or node positive or ductal grade 2–3 or hormone receptor negative) or not and allocated to one of four treatment regimens as follows.

A. Women at low risk or older than 70 years and hormone receptor negative post-menopausal women. No adjuvant treatment.
B. High risk pre-menopausal women with hormone receptor status positive or unknown.
  Treatment with chemotherapy (18 weeks) and Tamoxifen (5–10 years)
C. High risk post-menopausal women with hormone receptor status positive or unknown.
  Treatment with Tamoxifen (5–10 years)
D. High risk hormone receptor negative women younger than 70 years.
  Treatment with chemotherapy (18 weeks)

Furthermore, women were treated with radiotherapy if their surgical treatment was lumpectomy, the carcinoma was not removed micro-radically, or if age was < 70 years with positive nodes and tumour> 5 cm. These women were distributed in all four regimens and almost all women received radiotherapy.

In the later part of the recruitment period (from January 2007), the allocation to and content of treatment regimens changed slightly. The major changes were that, women were also categorised at high risk if they were lobular grade III or, Her2 positive or Top2A abnormal, only low risk women were assigned to no adjuvant treatment, and that Her2 positive women allocated to regimes B or D also were treated with Trastuzumab. Further details about history of the treatment regimens and allocation are available elsewhere [26].

Women not assigned a protocol, were not registered in DBCG, and thus information about adjuvant treatment was not available. These women were described separately.

The Danish National Patient Registry includes information on all hospital admissions (from 1977), emergency room visits and outpatient visits (from 1995) in both private and public hospitals in Denmark. Both a primary diagnosis and secondary diagnoses are registered along with information on procedures and treatments [27]. From this we obtained information about dates of mammography and of breast cancer diagnoses. We also calculated Charlson comorbidity index based on previous diagnoses in the registry [28]. The index was for analysis dichotomised in no comorbidity versus any number of comorbidities.

We identified four women, who were not diagnosed with breast cancer following the mammography at inclusion, but later. These were included in the group of women without cancer and excluded at their date of diagnosis.

The Danish Register for Evaluation of Marginalisation holds information on all public transfer payments administered by Danish ministries and municipalities for Danish citizens on a weekly basis since 1991. The type of transfer payment is recorded for each week if the person has received the benefit for 1 day or more. The registry contains more than 100 different codes for various past and present social transfer payments. Further information on the registry and its validity can be found elsewhere [29, 30]. We divided the women in four groups according to their type of income before mammography, namely Working, Temporary health-related benefits, Permanent health-related benefits and Retired at date of mammography. Age at mammography was categorized into 10-year age groups.

### Statistical analysis

We used restricted cubic splines to model the relationship between fatigue level and time, as we had no a priori hypotheses regarding the shape of these relationships. The data was analysed by mixed models with a random level for each woman. Time, centred at date of mammography, was introduced via cubic splines with 7 knots determined by percentiles (at − 18, 37, 179, 367, 651, 992, 1403 days) in the analyses of fatigue level and with 5 knots (at 55, 235, 504, 872 and 1354 days) in the analyses of the change in fatigue level from baseline. The choice of knot was decided based on plots of the curves with different number of knots and the maximum possible in Stata (7 knots), but in the analysis of fatigue, the curves were comparable using 5, 6 and 7 knots, so we chose 5 to obtain the most precise estimates. In analyses comparing different groups of women we introduced an interaction between the grouping variable and the cubic splines and the hypothesis of no interactions was tested by a Wald test. The analyses were made without adjustment as well as adjusted for the baseline characteristics, age groups (categorical; in 10 year groups), work status (categorical; working, temporarily health related benefit, permanent health related benefit and retirement pension), HADS score (linear; score from 0 to 42) and GH scores (linear; score from 0 to 100). Estimates are reported with 95% confidence intervals. All data management and analyses were done using Stata version 15.1.

## Results

Table 1 presents characteristic of the population, divided in women without breast cancer (or a later diagnosis) and women diagnosed with breast cancer as well as non-respondents with available information. Women who were diagnosed, more often suffered from comorbidity, were more often not working, were older, had slightly higher level of anxiety/depression and rated their general health lower than the women not diagnosed (Table 1). Non-respondents were a mix of women with and without breast cancer and their characteristics were consequently somewhere in between the characteristics of the participants with and without breast cancer (Table 1).

**Table 1:** Characteristics of women at the time of mammography categorized by cancer diagnosis

	All Count (%)	MFI fatigue score at baseline Mean (SD)	Not cancer Count (%)	Cancer Count (%)	Non-responders Count (%)
**Women**	323 (100)	28.8 (21.2)	204 (63)	119 (37)	2380 (100)
**Questionnaires**	2639 (100)		1425 (54)	1214 (46)	n/a
**Number of Questionnaires**					
2	25 (8)	33.5 (23.1)	19 (9)	6 (5)	n/a
3	20 (6)	35.3 (22.0)	17 (8)	3 (3)	n/a
4	78 (24)	28.2 (20.4)	72 (35)	6 (5)	n/a
5 to 14	168 (52)	28.5 (22.0)	68 (33)	100 (84)	n/a
15 to 17	32 (10)	24.2 (15.8)	28 (14)	4 (3)	n/a
**Days from mammography to BC diagnosis**					
N	123 (38)		4 (2)	119 (100)	142 (6)
Min	0		113	0	n/a
Max	1504		1504	40	n/a
Median	16		460	16	n/a
**Status at entry**					
**Comorbidity**					
None	289 (89)	27.3 (20.3)	189 (93)	100 (84)	2075 (87.2)
1	23 (7)	38.0 (24.9)	10 (5)	13 (11)	175 (7.4)
2 or more	11 (3)	49.9 (21.2)	5 (2)	6 (5)	130 (5.5)
**Work status**					
Working	205 (63)	24.9 (18.3)	156 (76)	49 (41)	1568 (65.9)
Health benefit - temporarily	32 (10)	35.6 (24.2)	15 (7)	17 (14)	259 (10.9)
Health benefit -permanently	65 (20)	35.6 (23.4)	32 (16)	33 (28)	411 (17.3)
Retirement pension	21 (7)	35.2 (26.5)	1 (0)	20 (17)	83 (3.5)
Missing					59 (2.6)
**DBCG-protocol**					
No protocol assigned	16 (5)	41.5 (25.7)	0 (0)	16 (13)	32 (1.3)
A	5 (2)	15.8 (18.7)	0 (0)	5 (4)	22 (0.9)
B	32 (10)	24.3 (22.4)	0 (0)	32 (27)	71 (3.0)
C	38 (12)	33.3 (24.2)	0 (0)	38 (32)	55 (2.3)
D	28 (9)	25.2 (19.1)	0 (0)	28 (24)	31 (1.3)
Irrelevant/missing	204 (63)	28.5 (20.0)	204 (100)	0 (0)	2239 (94.1)
**Age**					
Younger than 30	17 (5)	21.3 (13.9)	16 (8)	1 (1)	99 (4.2)
30–39	49 (15)	29.5 (22.5)	41 (20)	8 (7)	566 (23.8)
40–49	90 (28)	28.0 (21.3)	74 (36)	16 (13)	784 (32.9)
50–59	95 (29)	31.0 (20.3)	49 (24)	46 (39)	543 (22.8)
Older than 60	72 (22)	28.1 (22.7)	24 (12)	48 (40)	324 (13.6)
Missing					64 (2.7)
**HADS total score**					
N	320 (99)		201 (99)	119 (100)	n/a
Mean	11.16		10.88	11.62	n/a
SD	7.48		7.24	7.86	n/a
**GH-1 score**					
N	318 (98)		201 (99)	117 (98)	n/a
Mean	75.04		76.03	73.35	n/a
SD	20.78		18.99	23.53	n/a
**MFI fatigue score at baseline**					
N	323 (100)		204 (100)	119 (100)	n/a
Mean	28.79		28.48	29.33	n/a
SD	21.18		19.96	23.21	n/a

**Fig. 2:**
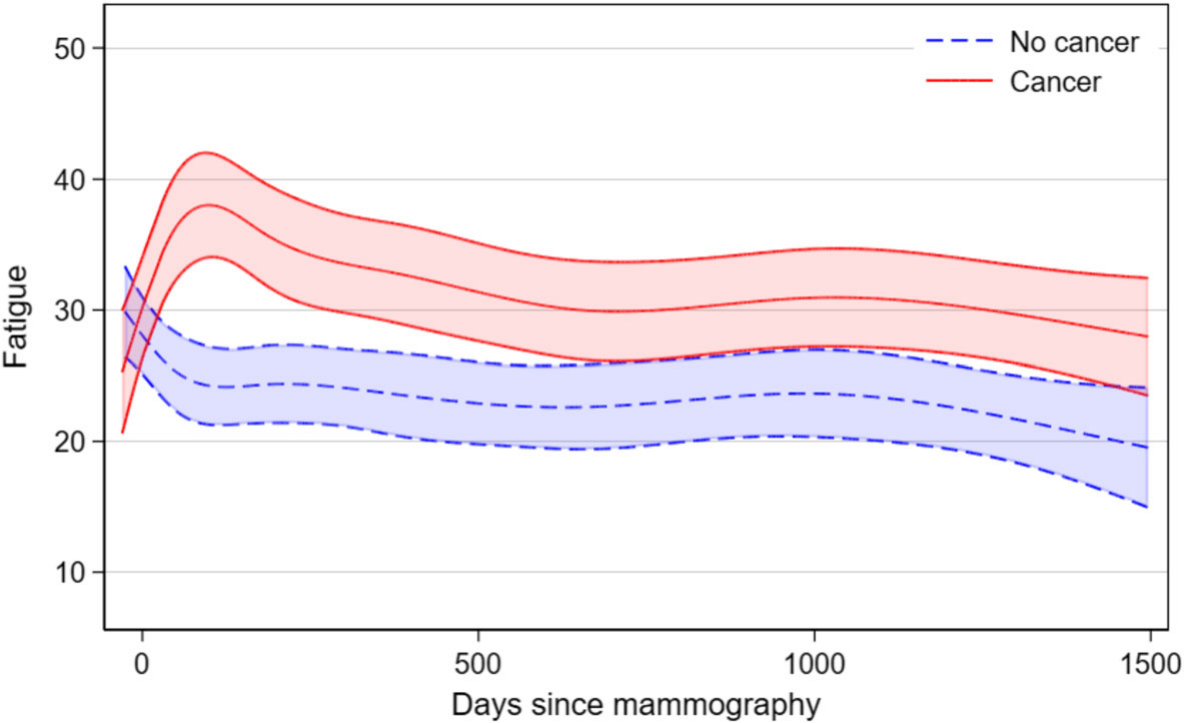
Mean fatigue in women with and without breast cancer

Figure 2 describes the course of fatigue. Women diagnosed with breast cancer are represented by the **solid** line, while women without breast cancer are represented by a dotted line, while shaded areas represent the 95% confidence intervals. Day 0 represents the date of mammography, reflecting that all women were unaware of if they were given the diagnosis after the mammography. The figure shows that women who are not diagnosed, are experiencing a decrease of fatigue followed by a steadier period, while women who were diagnosed, experience an increase in the months following the diagnosis, followed by a decrease over time in the following years.

**Fig. 3:**
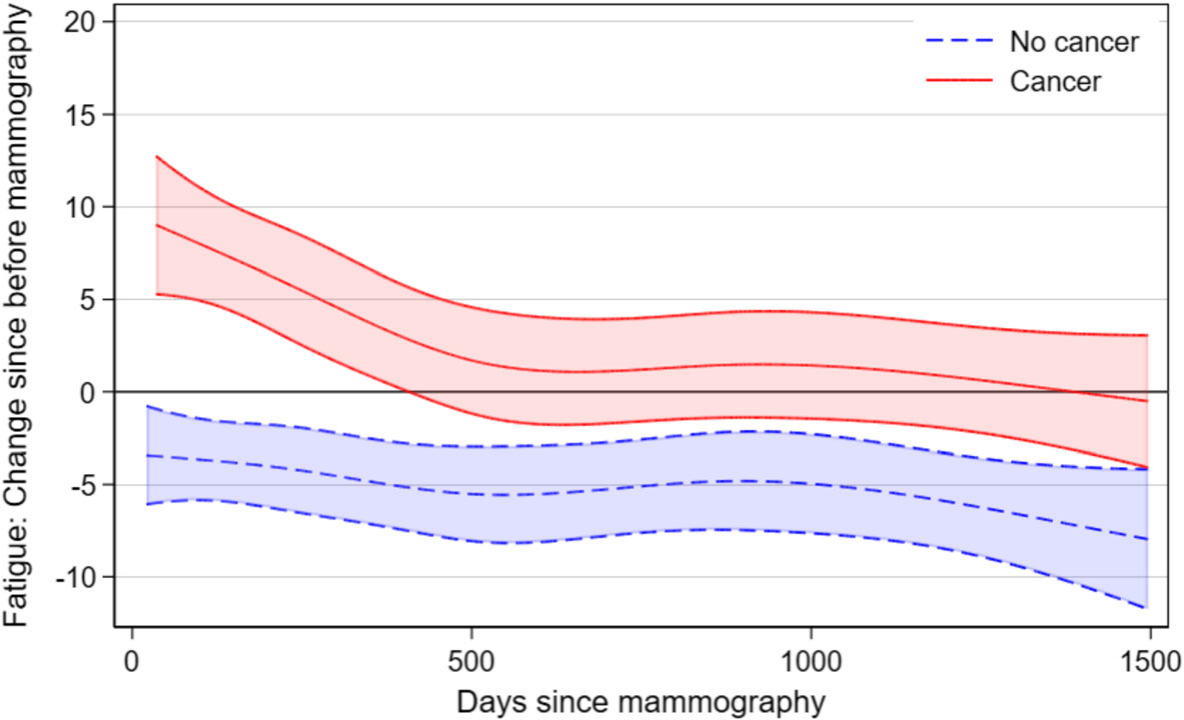
Mean change in fatigue from before mammography among women with and without breast cancer

When looking at the two groups in relation to their initial response in the questionnaire before the mammography, we found that women not diagnosed with breast experience a rapid relief from fatigue, while women diagnosed, are experiencing a large increase during the treatment period, followed by relief during the following year. Even after several years of follow up, the women with breast cancer consistently reported a higher level of fatigue, in comparison to women without the diagnosis (Fig. 3).

**Fig. 4:**
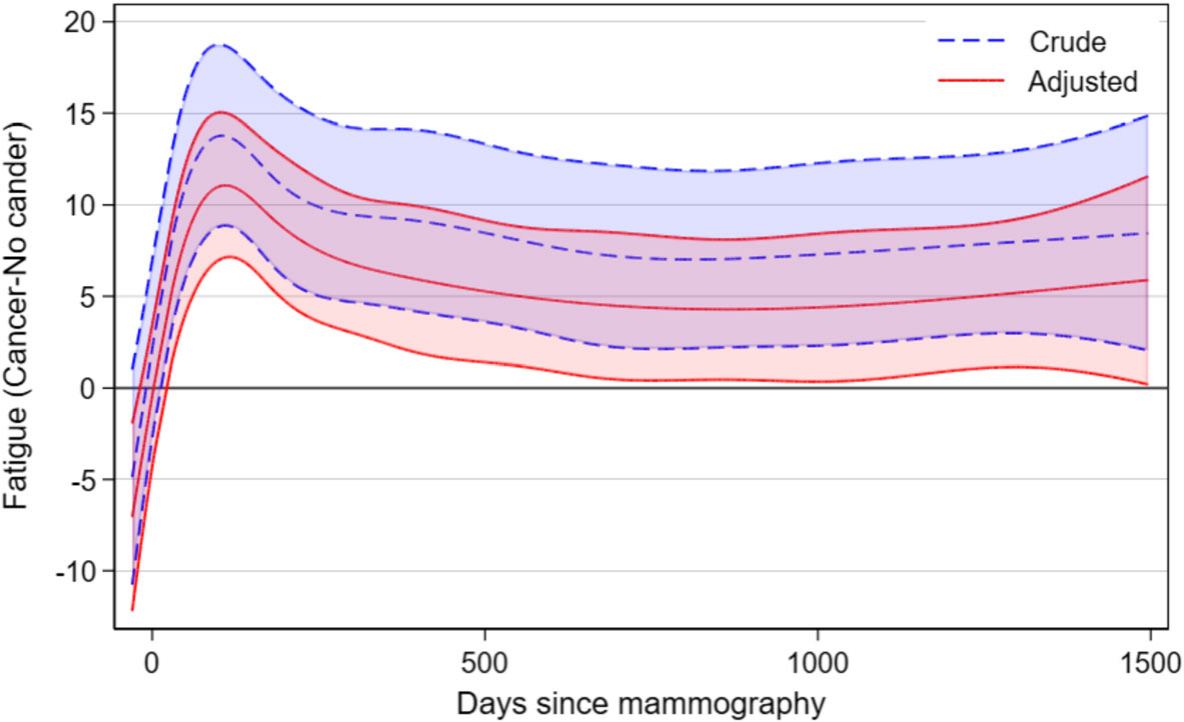
Change in fatigue comparing women with breast cancer to women without breast cancer

Figure 4 shows the difference in fatigue in women with breast cancer compared to women without breast cancer. An increase in the difference occurs in the first months following the diagnosis, where the treatment is most intense, followed by small decrease. The degree of fatigue then stabilised on a higher level among women with cancer. After adjustment, the difference between the two groups decreased, but the picture remained.

**Fig. 5:**
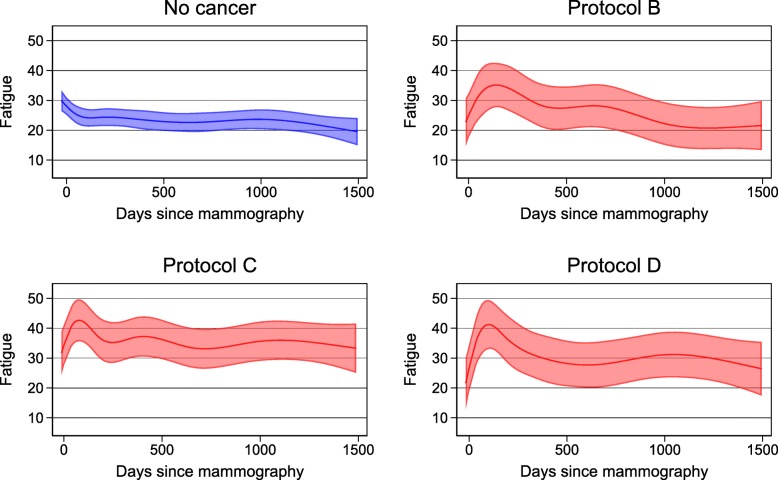
Mean fatigue among women with breast cancer divided according to treatment regimes

Figure 5 shows the course of fatigue in women with breast cancer according to the different treatment regimens compared to women without breast cancer. In general, the courses for the women with breast cancer are comparable. Women in regimen A (*n*=5) and women not assigned a regime (*n*=16) are not presented in Fig. 5 due to small strata in regimen A and heterogeneity in the women not assigned a regime.

**Fig. 6:**
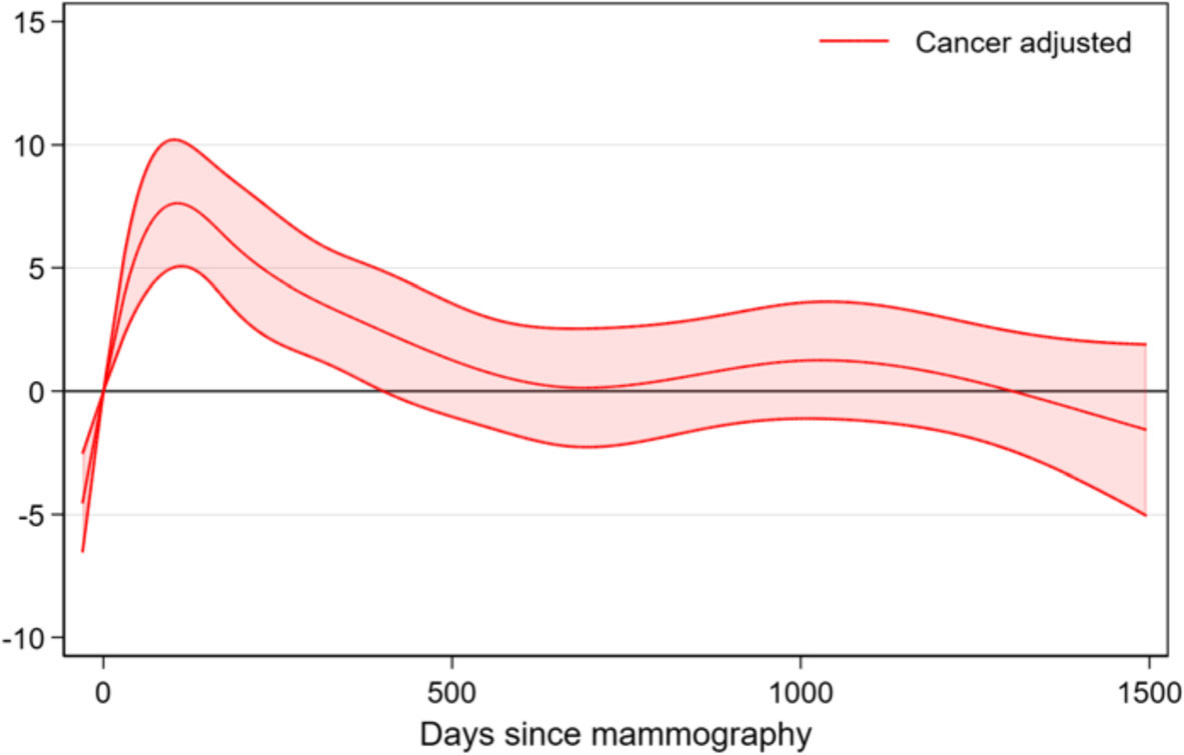
Course of fatigue related to fatigue level before mammography for women with breast cancer

Figure 6 shows fatigue among women with breast cancer compared to the level at mammography. There is a rapid increase in fatigue followed by a slow decrease, which levels off after approximately two years

## Discussion

The women with breast cancer reported a large increase of fatigue, especially in the first half a year or more with intense treatment, followed by a slow decrease over time. Despite the long follow-up period, the women with breast cancer did not return to their level of fatigue at time of the mammography. Women without breast cancer, on the other hand, reported a relief from fatigue in the same period, after their mammography did not show cancer and reached a steady level after that.

This study aimed to describe the course of fatigue in women diagnosed with breast cancer, starting before the diagnosis and to compare the course with women without breast cancer, but with the common characteristic, that all women were referred to mammography based on clinical suspicion of cancer. The women reported their level of fatigue before the mammography and thus without knowledge of whether they had cancer or not, but sharing the same worries. However, the true baseline level of fatigue was unknown for all women as their worries in relation to mammography may most likely have affected their well-being as we observe in the women without breast cancer. The mental impact of having a life-threatening illness may cause fatigue itself. Servaes et al. found that high anxiety, high impairment in role functioning and low sense of control over fatigue symptoms at baseline were predictors of persistent fatigue [31]. The magnitude of this effect may be quantified in the women without cancer, who presumably return to their habitual levels.

The different treatment regimens showed the same pattern, but the level of fatigue was highest in treatment regimen C, followed by treatment regimen B and D. Adjustment for age, comorbidity, work status, and anxiety/depression level and general health at baseline slightly decreased the level of fatigue.

This study’s important strengths were the register-based information regarding treatment, the inclusion of women before diagnosis with comparison to women without cancer, and the repeated and frequent follow-up of fatigue for a longer period. However, some important limitations must be considered when interpreting the results. First, the size of the study was not as large as intended, as a fast-track course was established for suspected cancers, so that it was no longer possible to recruit women before the mammography. Secondly, there are some risks of selection bias, both due to non-response and to attrition in the study. Since the outcome fatigue may be closely related to not answering the questionnaire, women who suffer the most from fatigue may be underrepresented in the later stages of the study. This may have caused an underestimation of fatigue; however, the pattern of the course is most likely the same. This may also explain the decrease in the level of fatigue in the end of the study, if those who have not left the study feel more vital than those who drop out. There was a tendency that the women reported a slightly higher level of fatigue, when answering the last questionnaire before attrition compared to the proceeding. This was the case for both women without breast cancer, but more pronounced for women with breast cancer (data not shown). This supports that the true course of fatigue may be less decreasing over time, than what the graphs shows and thus cause bias of unknown size due to attrition.

The relief we saw in the group of women without cancer shortly after the mammography may suggest that the reporting of fatigue is not solely related to treatment and symptoms, but also to the psychological distress related to the risk of suffering from a potential life-threatening disease, that both women with and without cancer experience. This is in line with the study from Servaes et al. who found that psychological distress was related to fatigue [31].

The DBCG register is considered valid and complete, however, the register has important limitations as well. Women, who have previously been treated for breast cancer, will not appear in the register, if they experience a contra-lateral breast cancer. Also, not all women are included in the database, if they are not eligible for inclusion in the standard treatment regimens, i.e. women with other life-threatening diseases. In the group of women referred to mammography and with a subsequent cancer diagnosis, 2 were not found in the DBCG database.

A few women answered the baseline questionnaire after the mammography, and thus may have had some knowledge about the result. We chose to exclude those (*n* = 11) that answered more than 3 days after the mammography, under the assumption that the remaining did not know their result, but we cannot be certain, since we did not have data on the date of receiving the result.

Our findings are in line with previous studies that reported that fatigue was a persistent problem among women with breast cancer [12, 16, 32, 33]. However, the recruitment procedures were not comparable, since we included women before diagnosis, while others included women after diagnosis and in some studies much later [16]. We have not been able to locate other studies of fatigue where women were enrolled before diagnosis. We identified one study with comparisons to women without breast cancer [33], but these were healthy controls and not with clinical suspicion of cancer, as in our study. Both women that are later diagnosed and women that do not suffer from breast cancer most likely share the same fatigue related to fear of having cancer. The course for fatigue for women with breast cancer in our study with increase in fatigue during first months of treatment were comparable to other studies [14, 32]. This does also reflect that radiotherapy often is used shortly after the diagnosis, adding further to the feeling of fatigue.

Only few previous studies applied repeated measurements, for example a study of breast cancer patients treated with radiotherapy, who were completing the Lee Fatigue Scale every 2 weeks for 2 months, and once a month for 2 months during and following radiotherapy [34]. Another study measured fatigue and other health related quality of life measures before diagnosis, 3 months after initial treatment and 1 year after completion of treatment, and found that elevated levels of fatigue was persistent 18 months after initial treatment [35], while a recent study measured fatigue before treatment and after four and 8 months [32], a study from Taiwan measured fatigue 9 times during the first year after treatment [36] and finally a recent French study with 10 measurement with MFI-20 during 2 years after diagnosis [12]. The latter study does not compare to women without cancer and starts follow-up after diagnosis. Furthermore the different types of treatments were not taken into account [12]. An American study described trajectories of fatigue for up to 4 years, but this study started after the treatment period [17] .Thus this current study is to our knowledge the first with a combination of long-term follow-up and many measurements and with comparison to women without cancer and over different treatment regimens.

The findings of the study can be generalised to women with breast cancer, where the referral to mammography are based on reporting of symptoms, and not from a screening programme. However, the course of fatigue is most likely comparable to all women under treatment following breast cancer. Regimens of cancer treatment are in constant development with fatigue as a well-known side-effect to namely chemotherapy, meaning that the symptoms reported during treatment a decade ago may not precisely reflect women’s symptoms today.

## Conclusion

Fatigue is a persistent problem in women diagnosed with breast cancer, even several years following diagnosis and treatment. The women with cancer seem most affected during the first 6 months after diagnosis, related to the period of most intensive treatment. Even women, who are not diagnosed with cancer, report fatigue before they have had confirmation that they do not suffer from cancer diagnosis, where after the fatigue disappears.

## Data Availability

The data that support the findings of this study are available from the authors, DBCG, The Danish National Patient Registry and Danish Register for Evaluation of Marginalisation. Restrictions apply to the availability of these data, which were used under license for this study.

## Abbreviations

MFI-20: The Multidimensional Fatigue Inventory
DBCG: Danish Breast Cancer Cooperative Group
GH: The General Health domain in Short-Form-36
HADS: The Hospital Anxiety and Depression Scale
